# Antitumor effect of Quercetin on Y79 retinoblastoma cells via activation of JNK and p38 MAPK pathways

**DOI:** 10.1186/s12906-017-2023-6

**Published:** 2017-12-13

**Authors:** Haojie Liu, Ming Zhou

**Affiliations:** Department of Ophthalmology, Affiliated zhongshan hospital of dalian university, Jiefang Road 6, Dalian, Liao Ning 116001 China

**Keywords:** Quercetin, Retinoblastoma, Apoptosis, c-Jun N-terminal kinase, p38 mitogen-activated protein kinase

## Abstract

**Background:**

Quercetin (QCT) is a flavonol present in many vegetables, it is proved to show chemo preventive effect against lung, cervical, prostate, breast and colon cancer due to its anti-inflammatory, anti-tumor and anti-oxidant property. Looking into the reported chemo-preventive effect we speculated antitumor activity in retinoblastoma (RB) Y79 cells, we also studied the molecular mechanism for antitumor activity.

**Methods:**

The effect of QCT on Y79 cell viability count was done by cell counting kit, cell cycle distribution, apoptosis studies and mitochondrial membrane potential was evaluated by flow cytometry. Protein expression was done by western blot analysis.

**Results:**

The outcomes of study showed that QCT reduced Y79 cell viability and caused arrest of G1 phase in cell cycle via decreasing the expression levels of cyclin-dependent kinase (CDK)2/6 and cyclin D3 and by increasing the levels of both CDK inhibitor proteins p21 and p27. Apoptosis of Y79 cells mediated by QCT occurred via activation of both caspases-3/-9. Flow cytometry studies showed that QCT caused collapse in mitochondrial membrane potential (ΔΨm) in Y79 cells. Western blot studies confirmed that QCT brought about phosphorylation of c-Jun N-terminal kinase (JNK) and p38 mitogen-activated protein kinase (MAPK). We also established that inhibitors of JNK and p38 MAPK suppressed QCT mediated activation of both caspases-3/-9 and subdued the apoptosis of cancerous Y79 cells.

**Conclusion:**

All the results of the study suggest that QCT induced the apoptosis of Y79 cells via activation of JNK and p38 MAPK pathways, providing a novel treatment approach for human RB.

## Background

Retinoblastoma (RB) has been reported to be one of the most occurring malignancies of intraocular cavity in small children’s. It has been reported to affect about every 1 in 15,000 children worldwide [1]. The etiology of the disease is associated with alterations in the RB tumour suppressor gene (RB1) of the retinal progenitor cells. It has also been documented that children’s who suffer with the heritable form of RB inherit with one of the mutated RB1 allele and in the process of developing retinal cells loss of the other allele occurs somatically. Such children’s often acquire bilateral and multifocal tumors of retina which further leads to development of some other primary tumors of bones or soft tissues in later part of their life [2]. The treatment options for RB consist of chemo-reduction by intravenous infusions, transpupillary thermotherapy, laser guided photocoagulation, orbital exenteration and radiotherapy and chemotherapy. All these treatment options are selected on the basis of location, size and stage of development of RB tumor [3, 4]. All of these multiple treatment options are associated with unwanted clinical side effects such as infection, gastrointestinal damage, fever and in extreme can cause blindness [5]. Therefore, there is a pressing demand for the development of new therapeutic agents for treating RB.

Quercetin (3,5,7,3′,4′-pentahydroxyflavone) (QCT) is a flavonol present in many vegetables which is a subclass of flavonoid [6]. It can interact with a broad range of enzymes specifically receptor kinases, protein kinase C, cyclin-dependent kinases (Cdks) and also with MEK-ERK signaling [7]. QCT is established as a chemo preventive agent in many types of cancers such as lung, cervical, prostate, breast and colon due to its anti-inflammatory, anti-tumor and anti-oxidant property [8]. Number of mechanisms for its anti proliferative action are established which include 1. Apoptosis by cell cycle arrest [9] 2. Arrest of phase G_2_/M or G_1_ [10] 3. Disruption of mitochondria and microtubules 4. Production of stress proteins 5. Activation of caspases and 6. Release of cytochrome C [11]. In addition to this it has also been found that QCT can chelate metals, scavenge oxygen free radicals, inhibit xanthine oxidase and lipid peroxidation in vitro due to its antioxidant activity [12–16].

However there are no reports exploring effects of QCT in treatment of RB, in the present study we evaluated the molecular mechanism responsible for QCT-induced alterations in human Y79 RB cells.

## Methods

### Cell lines and culture conditions

The cancerous Y79 human RB cells were obtained from St. Jude Children’s Res. Hosp (TN). The cancerous RB Y79 cells were cultured in RPMI-1640 medium consisting fetal bovine serum (FBS) (10%), penicillin (1%) and streptomycin, followed by storage at 37 °C in 5% CO_2_.

### Reagents and antibodies

Quercetin was obtained from Sigma-Aldrich (U.S.A), ZVAD-FMK, antibodies against p21, cytochrome c, GAPDH, caspase-9, caspase-3, cyclin D3 and p27 KIP1 were obtained from Abcam, UK. Antibodies for c-Jun N-terminal kinase (JNK), phospho-JNK, p38 MAPK, cyclin-dependent kinase-2 (CDK-2), cyclin-dependent kinase-6 (CDK-6) and phospho-p38 MAPK were received from Cell Signaling Technology, U.S.A.

### Cell treatment

Quercetin for all the experiments was prepared by dissolving in dimethylsulfoxide (DMSO) from Sigma-Aldrich, USA, prepared freshly every time before any treatment. The prepared DMSO stock solution of QCT was used to make final defined concentrations (25-100 μM) in culture medium [17]. controls were prepared by preparing 0.07% solution of DMSO in culture media in all the experiments. The Y79 cells were subjected to ZVAD-FMK (50 μM) for 60 min to inhibit the caspase activity. The Y79 cells were also treated with and without JNK inhibitor (20 μM) / MAPK inhibitor p38 for 60 min followed by treatment of Quercitin (100 μM) for 24 h prior to evaluating phosphorylation levels of JNK and p38 MAPK.

### Cell viability studies

The cell viability count was done by cell counting kit (CCK; Sigma-Aldrich, U.S.A). Briefly, the Y79 cells at density of 5 × 10^3^/well were incubated along with QCT added RAPMI medium in plates containing 96-wells for 24 h followed by replacement of culture medium with fresh medium containing 10 ml CCK-8 solution. The cells were incubated at 37 °C for 2 h. the viability was calculated by recording optical density (OD) at 450 nm.

### Flow cytometry for cell cycle analysis

The Y79 cells were subjected to seeding for overnight in 10% FBS culture medium in 6 well culture plates at a density of 4 × 10^5^ followed by treatment with various concentrations of QCT (0-100 μM) for 24 h. The cell cycle studies were done, briefly, the cells were treated with propidium iodide (PI) solution (0.5 ml) followed by incubation of 30 min (all the procedure in accordance to manufacturer’s instructions). The analysis of cell cycle distribution was done by flow cytometry (Bio-Rad, USA).

### Apoptosis studies by flow cytometry

Extent of apoptosis was evaluated by Annexin-V- FITC staining using an Annexin V-fluorescein isothiocyanate apoptosis detection kit (Bio-Rad; USA) as per the manufacturer’s instructions.

### Western blot analysis

For western blot analysis the Y79 cells were subjected to lyses at 4 °C for 30 min in a buffer solution Tris buffer (50 mM, pH 8.0), sodium chloride (150 mM), EDTA (5 mM), Nonidet p-40 (1% *v*/v), aprotinin (20 g/mL), phenylmethylsulfonyl fluoride (1 mM) and leupeptin (25 g/mL). The protein extracts were determined using Pierce™ SDS-PAGE Sample Prep Kit (Thermo Scientific™), followed by transfer to a polyvinylidene difluoride (PVDF) membrane. The obtained fraction was then treated with Tris-HCl buffer (20 mM, pH 7.5), sodium chloride (500 mM), non-fat milk (5%) for 2 h at room temperature conditions followed by incubation with specific antibody overnight at 4 °C, the blots were imaged using Immobilon Western Chemiluminescent HRP Substrate (Merck Millipore).

### Determination of mitochondrial membrane potential (MMP) (ΔΨm)

In the method the cells were subjected to seeding in a 6-well containing plate and were treated with varying concentrations of QCT for 24 h. The cells were washed with PBS and were stained with Rh-123 (1 μg/ml) for 30 min in the dark at 37 °C. For the study mean fluorescence intensity (MFI) of treated cells was by peforming flow cytometric analysis.

### Statistical analysis

All the data are presented as the means ± standard deviation (SD). Comparison between results was done by one-way ANOVA followed by Dunnett’s test with Graphpad-prism-7 software. Value of *P* < 0.05 was looked at to be a value of statistically significant.

## Results

### Quercetin inhibited cell viability in Y79 cells

**Fig. 1:**
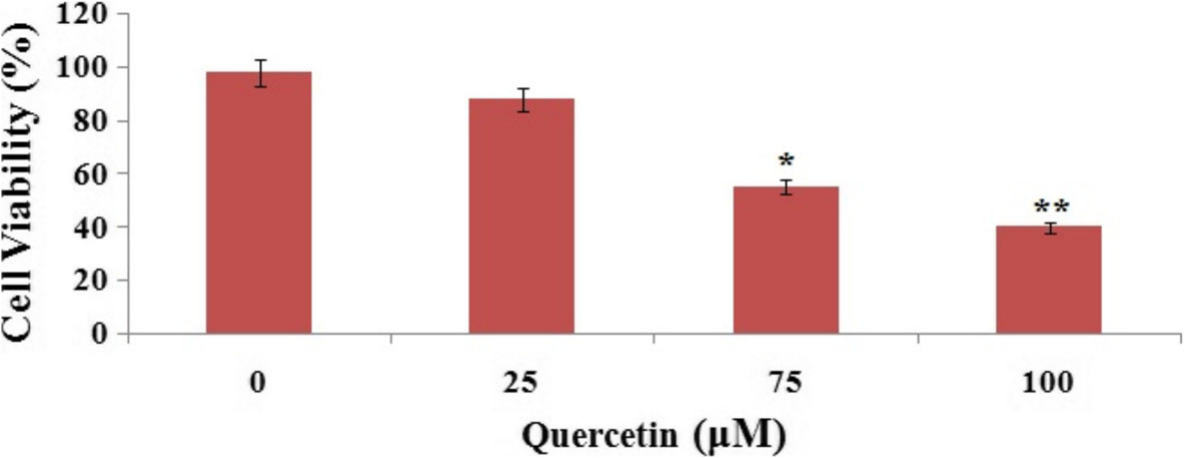
Study of various concentration of Quercetin on the viability of Y79 human retinoblastoma cells in vitro. Cell viability was evaluated at various concentrations of Quercetin (0-100 μM) for 24 h. All the data are means ± SD (*n* = 3) and are represented as percentage of control cells. *P < 0.05,**P < 0.01 compared to control

Cell viability study was done in vitro by treating RB Y79 cells with different concentrations of QCT (0, 25, 75, 100 μM) for 24 h. The changes in Y79 cell viability were marked by performing CCK-8 assay. As depicted in Fig. 1, the cell viability decreased with increasing concentration of QCT compared to control cells, the results were highly significant with concentration of 100 μM (*P* < 0.01).

### Quercetin causes cell cycle arrest in Y79 RB cells

**Fig. 2:**
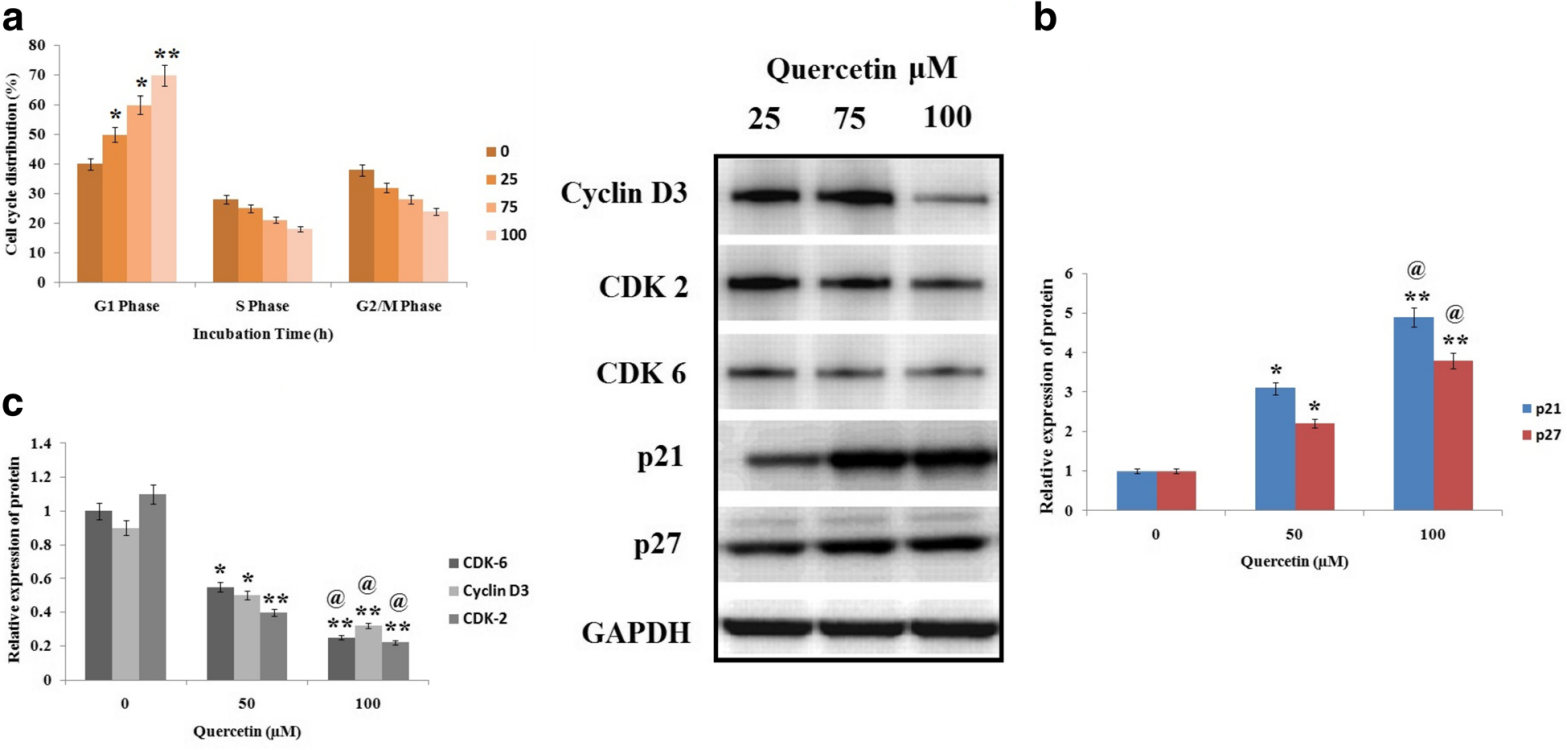
Quercetin causes arrest of cell cycle in retinoblastoma (RB) Y79 cells in vitro. **a** distribution of cell cycle of RB Y79 cells after 24 h of treatment with different concentrations of Quercetin (0-100 μM), all data are represented as the means ± SD (*n* = 3). *P < 0.05, **P < 0.01 against control. **b** and **c** Y79 cells were exposed to various concentration of Quercetin (0-100 μM) and the obtained cell lysates after 24 h were subjected to western blot analysis employing specific antibodies against cyclin-dependent kinase (CDK)2, CDK6, p27, cyclin D3 and p21, GAPDH was used as a loading control. The results are represented as means ± SD (*n* = 3). *P < 0.05, **P < 0.01 against control. @P < 0.05 against Quercetin (50 μM) group

Effect of QCT on cell cycle progression in Y79 RB cells was analyzed, in the process cells were treated with QCT at defined concentrations (0, 25,75,100 μM) for 24 h. The cells after treatments were analyzed by flow cytometry for marking any alterations. As per the results (Fig. 2a), QCT treated RB cells after 24 h showed signs of inhibition in G1 phase compared to S and G2/M phase. The molecular mechanism responsible for G1 phase arrest was assessed by analyzing the expression of various proteins by western blot analysis in Y79 cells treated with QCT (0, 50 and 100 μM). On western blot studies the results (Fig. 2b and c) suggested marked reduction in expression of CDK6, CDK2 and cyclin D3 compared to control group cells, whereas the expression levels of p27 and p21 which are CDK inhibitor proteins increased after treating RB cells to QCT. The expressions of these proteins suggest involvement of cyclin-CDK pathway in QCT mediated G1 phase arrest of cell cycle in Y79 RB cells.

### Quercetin causes apoptosis of Y79 RB cells

**Fig. 3:**
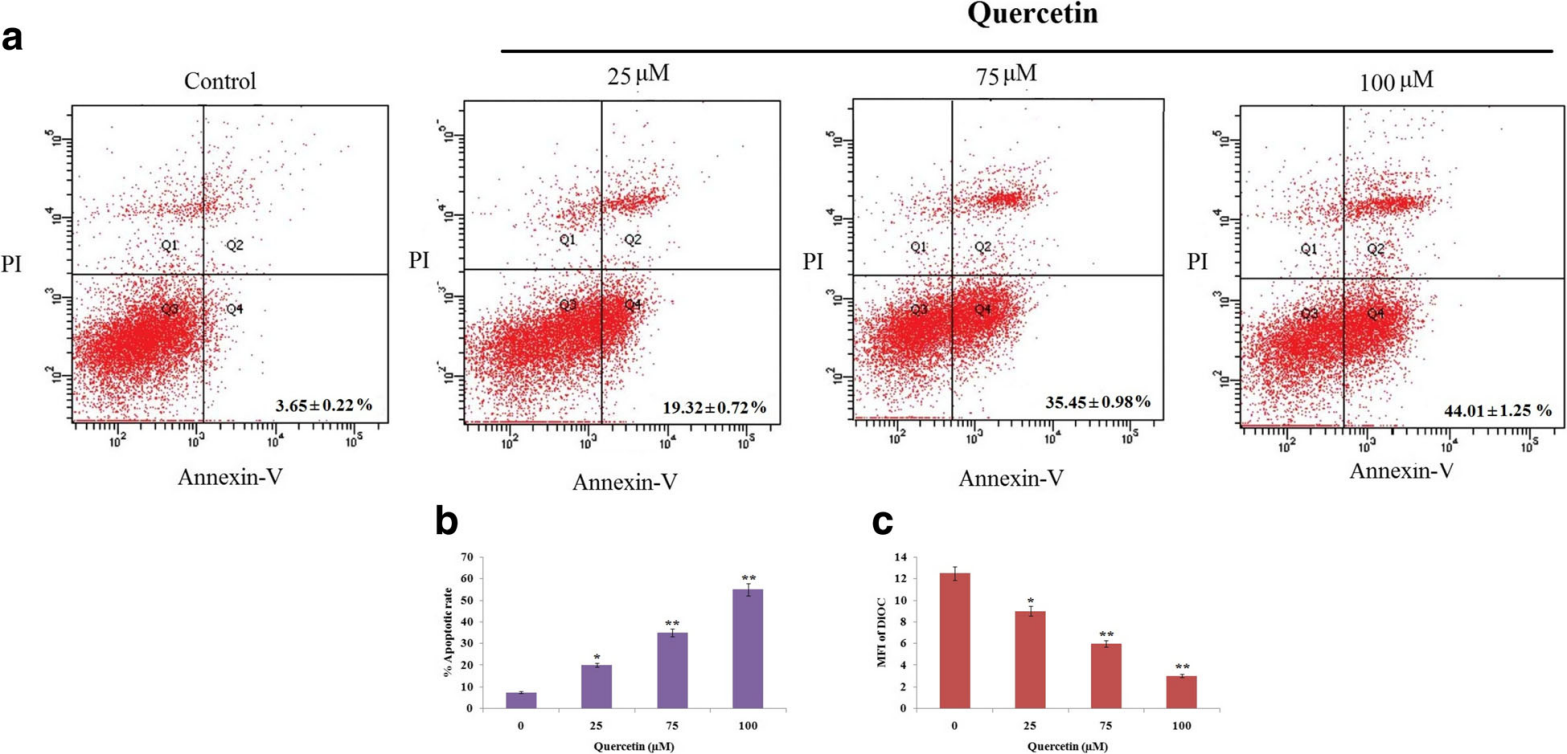
Quercetin causes apoptosis of RB Y79 cells in vitro. **a** The control cells (untreated), the treated cells received various concentration of Quercetin (0-100 μM) for 24 h. Extent of apoptotic cells were assessed by flow cytometry subjecting cells to staining of propidium iodide (PI) and Annexin V-fluorescein isothiocyanate (FITC). **b** Results represent analysis of the total apoptotic cell population. All the data presented are the means ± SD (*n* = 3). *P < 0.05, **P < 0.01 against control. **c** Quercetin caused ΔΨm collapse in cancerous RB Y79 cells. Flow cytometric analysis was done in order to record the mean fluorescence intensity (MFI) of DiOC_6_. All the values are the means ± SD (*n* = 3). *P < 0.05, **P < 0.01 against control

In process to evaluate involvement of apoptosis in cell viability of Y79 RB cells, we treated Y79 RB cells with varied concentrations of QCT (0, 25, 75, 100 μM) for 24 h followed by Annexin-V and Propidium Iodide (PI) assay. The results (Fig. 3a and b) suggested that the % PI negative/ Annexin V positive and PI-positive/Annexin V-positive were 3.65% in control and 19.32, 35.45, 44.01% for Y79 cells treated with QCT 25, 75 and 100 μM respectively. Further in order to analyze the effect of QCT on mitochondria of Y79 cells, the cells treated with QCT 9 (0, 25, 75, 100 μM) for 24 h were exposed to DiOC6 dye. The DiOC6 is a cationic lipophilic dye which accumulates specifically in the mitochondria as a property of its mitochondrial membrane potential (ΔΨm) which is reported to decrease in cells undergoing apoptosis [18, 19]. The DiOC6 treated cells were analyzed for mean fluorescence intensity (MFI) using flow cytometry. The results of the experiment suggested a significant decrease in MFI of DiOC6 (Fig. 3c) in cells treated with QCT, highly significant results were seen in cells treated with 50 and 100 μM of QCT. Overall the results suggested that treatment of QCT induced apoptosis may be due to destruction of mitochondrial membrane potential in cancerous Y79 cells.

### Quercetin induces the apoptosis via activation of caspases in RB Y79 cells

**Fig. 4:**
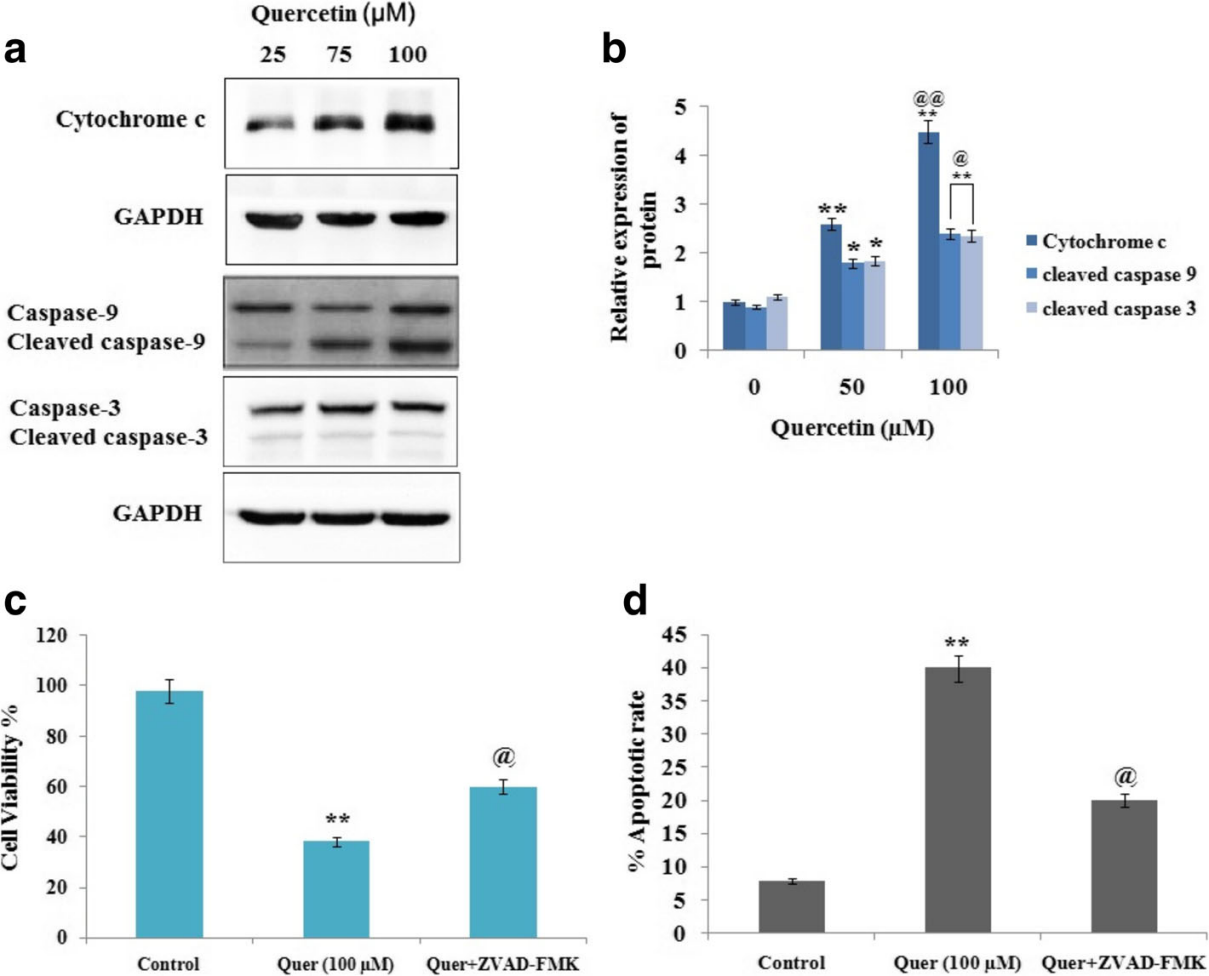
Quercetin causes apoptosis of cancerous RB Y79 cells via intrinsic pathways. **a** and **b** The Y79 cells were exposed to Quercetin (0-100 μM). The obtained cell lysates after 24 h were analyzed by western blot using specific antibodies against caspase-9, caspase-3 and cytochrome *c*, GAPDH was used as a loading control. *P < 0.05, **P < 0.01 against control (*n* = 3). @*P* < 0.05, @@P < 0.01 against Quercetin 50 μM. **c** Y79 cells were exposed to 100 μM Quercetin for 24 h in the presence or absence of ZVAD-FMK. Viability of cells was done by the Cell Counting Kit-8, the data are presented as the percentage of control cells, means ± SD (*n* = 3), **P < 0.01 against control, @P < 0.05 against Quercetin treated group. **d** Quercetin exposed apoptosis was blocked by ZVAD-FMK treatment, the data presented are means ± SD (*n* = 3). **P < 0.01 against control, @*P* < 0.05 against Quercetin treated group

Mitochondrial membrane potential (MMP) is a important factor in regulating cellular functions, any disturbances lead to alterations in membrane dynamics in response causing release of cytochrome *c*, which further leads to activation of caspase-9. In order to find whether QCT causes apoptosis mediated by release of cytochrome *c* and caspase-9, the Y79 RB cells were treated with defined concentrations of QCT (0, 50 and 100 μM) for 24 h. The cells extracts were subjected to western blot to analyze the expression levels of caspase-9. The results of blots suggested (Fig. 4a and b) that QCT resulted in increased levels of cytochrome *c* with subsequent activation of caspase-3 and caspase-9 (Fig. 4b) with increasing doses. Further, a pan-caspase inhibitor ZVAD-FMK was used to study the effects of QCT on apoptosis of Y79 cells. Results suggested (Fig. 4c), pre treatment of the pan-caspase inhibitor (ZVAD-FMK) had attenuating effect on QCT induced decrease in Y79 viability. Results also suggested that the pan-caspase inhibitor attenuated the QCT mediated apoptotic effect on Y79 RB cells. Overall the outcomes of experiment suggested involvement of caspase activation in QCT mediated apoptosis of RB Y79 cells (Fig. 4d).

### Role of JNK and p38 MAPK signaling pathway in activation of caspase-3 and -9 induced by Quercetin

**Fig. 5:**
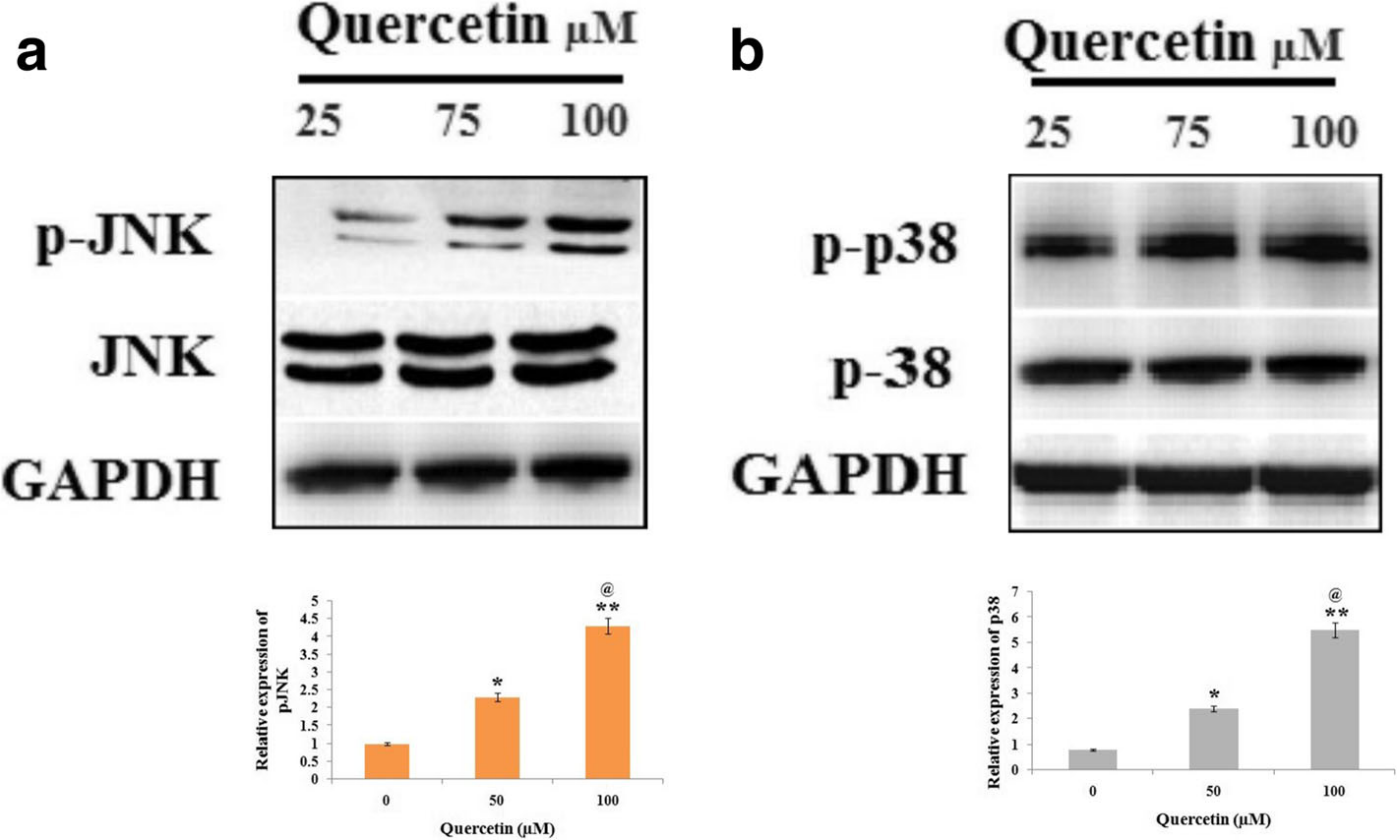
Quercetin causes phosphorylation of c-Jun N-terminal kinase (JNK) and p38 mitogen-activated protein kinase (MAPK) in RB Y79 cells. **a** and **b** The RB Y79 cells were treated with various concentrations of Quercetin (0-100 μM) and incubated for 24 h. The levels of phosphorylated (p-)JNK and p38 MAPK were assessed by western blot, GAPDH was used as a loading control. *P < 0.05, **P < 0.01 against control. @*P* < 0.05 against Quercetin (50 μM) (n = 3)

**Fig. 6:**
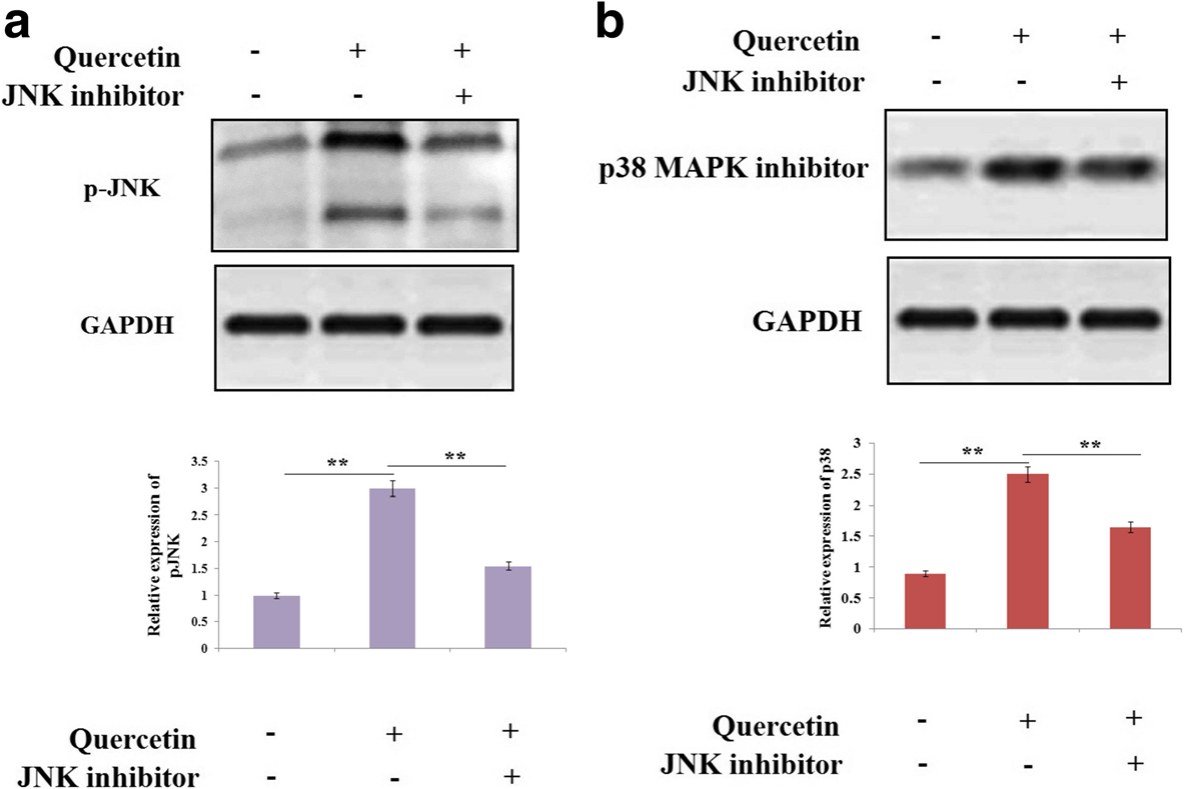
Study suggesting effect of JNK and MAPK inhibitors on Quercetin-mediated activation of JNK and p38 MAPK. **a** and **b** The RB Y79 cells were exposed in the presence or absence of JNK inhibitor and p38 MAPK inhibitor for time period of 1 h followed by treatment with 100 μM Quercetin for 24 h. The levels of phosphorylation of JNK and p38 MAPK were evaluated opting western blot analysis, in which GAPDH was opted as loading control. **P < 0.01 (n = 3)

**Fig. 7:**
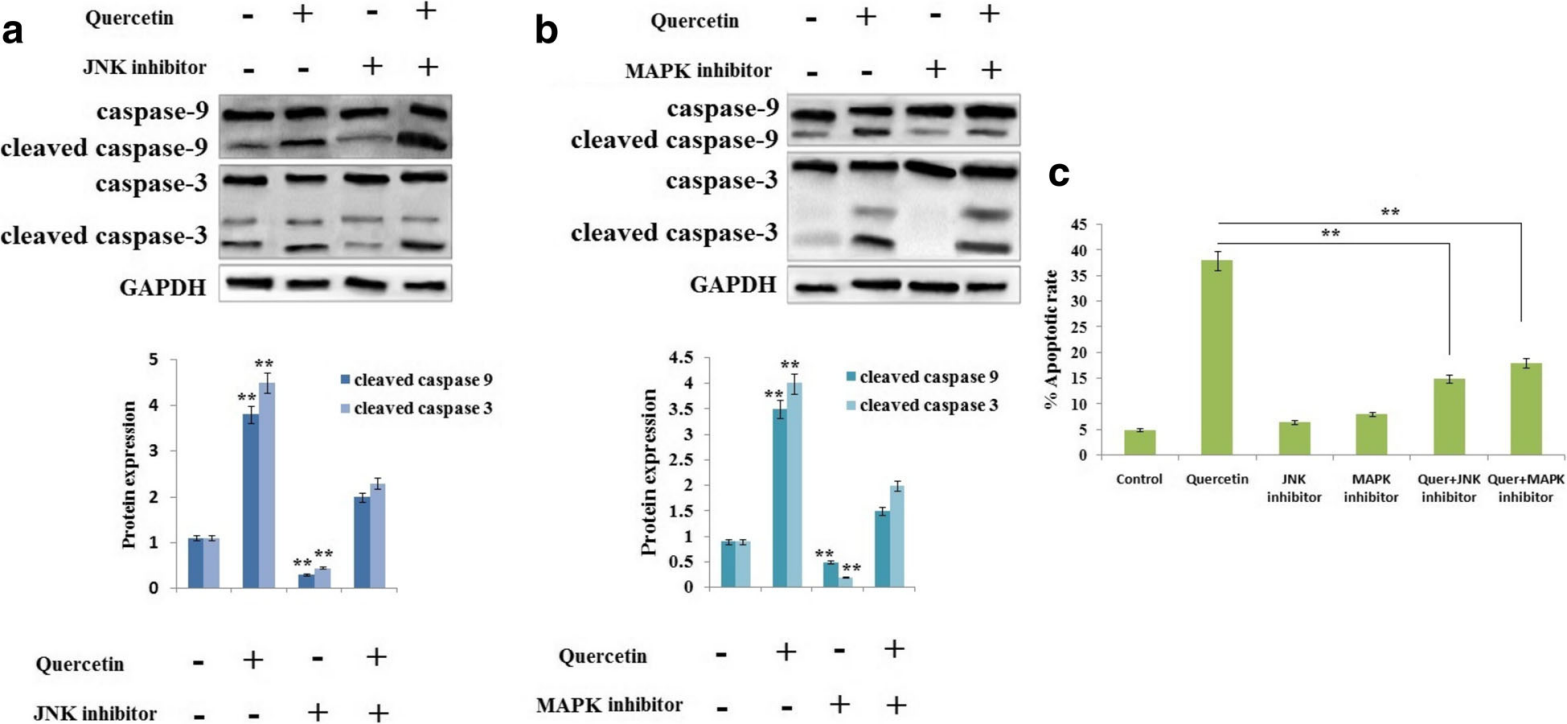
Effect of Quercetin induced apoptosis in RB Y79 cells mediated by JNK and p38 MAPK. **a** and **b** The RB Y79 cells were exposed in the presence or absence of JNK inhibitor or MAPK inhibitor for 1 h followed by treatment with 100 μM Quercetin for time period 24 h. Western blot analysis was done for levels of caspase-9 and -3, GAPDH was loading control. **P < 0.01 against control (n = 3). **c** Total apoptotic population was analyzed using flow cytometry with staining of propidium iodide (PI) and Annexin V-fluorescein isothiocyanate (FITC). The data represented as means ± SD (n = 3), **P < 0.01

In this experiment, we evaluated the QCT treated Y79 cells for activation of JNK and p38 MAPK by western blot analysis. The results suggested (Fig. 5) that QCT resulted in increased phosphorylation of JNK and p38 MAPK in RB cells. Further in order to evaluate status of JNK and p38 MAPK in QCT mediated apoptosis, the Y79 cells were exposed to JNK-inhibitor or p38 MAPK inhibitor for 60 min followed by treatment of QCT (100 μM) for 24 h. The Y79 cell extracts were subjected to western blot studies for expression of proteins, as found the blots (Fig. 6) suggested QCT suppressed the activation of JNK along with p38 MAPK. The experiment also suggested significant reduction in QCT mediated apoptosis (Fig. 7c) and activation of caspase-3/−9 (Fig. 7a and b). The experimental outcomes suggest QCT mediated activation of caspase-3 and caspase-9 may be due to activation of JNK and p38 MAPK.

## Discussion

Retinoblastoma is one of the most occurring intraocular cancer [20]. The treatment options available for managing RB include the chemotherapy which suffers disadvantage such as toxicity and other side effects, but still the survival rates are low in many developing countries [21, 22], hence becomes necessary to explore new drug molecules. QCT, a natural flavonol found in many vegetables, exerts chemo preventive action on many cancer types, such as lung, cervical, prostate, breast and colon. Number of mechanisms suggested its potential as an anticancer agent [7–16]. Although the anticancer effect of QCT is established for number of cancers, role in Retinoblastoma has not been explored. In present research work, we evaluated the potential of QCT to be used as anticancer agent to treat RB and explored the mechanism involved by treating it with Y79 RB cells.

Results of our study outlined that QCT decreased cell viability of RB Y79 cells with increasing dose. The cell proliferation was found to mediate via regulation of cell cycle. The cell cycle is regulated by number of process, Cyclin-dependent kinases (CDKs) are activated by cyclin regulatory subunits forming complex with them [23, 24]. Results of our study suggested QCT treatment resulted in accumulation of RB cells in g1 phase of cell cycle. We also found that treatment of QCT decreased the expression of proteins such as CDK6, cyclin D3, and CDK2, increase in expression of p27 and p21 which are CDK inhibitor proteins was observed suggesting involvement of these proteins in QCT mediated arrest of G1 phase of cell cycle in Y79 RB cells.

Apoptosis is a mechanism responsible for programmed death of cells [25]. Mitochondrial membrane potential (MMP) plays an important role in activation of caspase-9 via release of cytochrome *c* [26]. Literature confirm leading role of caspase-9 and caspase-3 in apoptosis [27, 28]. Outcomes of our study revealed that Quercetin caused increase in MMP leading to activation of caspase-dependent apoptotic pathway of mitochondria. Also we confirmed involvement of caspase-9 and caspase-3 in apoptosis, by treating Y79 cells with a pan-caspase inhibitor ZVAD-FMK followed by exposing them to QCT. Experiments were carried to evaluate role of JNK and p38 MAPK pathways in Querectin mediated apoptosis of Y79 RB cells. Results suggested QCT resulted in activation of JNK and p38 MAPK in cancerous Y79 cells. The activation of caspase-9 and caspase-3 was suppressed in Y79 cells treated with JNK and p38 MAPK inhibitor leading to decrease in Querectin-mediated apoptosis. Overall the results directed involvement of JNK and p38 MAPK pathways in Querectin mediated apoptosis of Y79 RB cells by regulating expressions of caspase-9/−3.

## Conclusion

In conclusion, the present research confirmed that QCT exerted anticancer effect on RB Y79 cells by inducing apoptosis and cell cycle arrest. These findings propose a novel therapeutic approach for treatment of RB which needs further clinical investigation.

## Abbreviations

CDK: Cyclin-dependent kinase
JNK: c-Jun N-terminal kinase
MAPK: p38 mitogen-activated protein kinase
QCT: Quercetin
RB: Retinoblastoma
